# LncRNA AC040162.3 Promotes HCV-Induced T2DM Deterioration through the miRNA-223-3p/NLRP3 Molecular Axis

**DOI:** 10.1155/2023/5350999

**Published:** 2023-06-17

**Authors:** Ben Niu, Xueshan Xia, Lijing Ma, Lixuan Yao, Yating Zhang, Heng Su

**Affiliations:** ^1^Department of Endocrinology and Metabolism, The First People's Hospital of Yunnan Province, The Affiliated Hospital of Kunming University of Science and Technology, Kunming, Yunnan, China; ^2^Faculty of Life Science and Technology, Kunming University of Science and Technology, Kunming, Yunnan, China

## Abstract

**Background:**

Diabetes is one of the most common diseases and major public health burdens worldwide. Type 2 diabetes mellitus (T2DM) is associated with chronic hepatitis C virus (HCV) infection, and lncRNAs play an important role in HCV-induced T2DM. We aimed to explore the effect of lncRNA AC040162.3 on HCV-induced T2DM.

**Methods:**

HCV was used to infect MIN6 cells to establish an in vitro model. HCV copy number and miRNA expression were detected by Real Time Quantitative PCR (RT-qPCR). Enzyme-Linked Immunosorbent Assay (ELISA) was used to detect the secretion of insulin, and methyl thiazolyl tetrazolium (MTT) was applied to analyze cell viability. Apoptosis was analyzed by Western blotting and flow cytometry. In addition, Western blotting and TdT-mediated dUTP Nick End Labeling (TUNEL) were used to analyze pyroptosis. Luciferase reporter assays were used to investigate the targeting relationship.

**Results:**

The expression of LncRNA AC040162.3 and NLRP3 was markedly increased in HCV–T2DM, while the expression of miR-223-3p was remarkably inhibited. In vitro experiments demonstrated that lncRNA AC040162.3 silencing or miR-223-3p overexpression remarkably alleviated HCV-induced T2DM deterioration by inhibiting cell apoptosis and pyroptosis and enhancing cell viability. We then demonstrated that silencing lncRNA AC040162.3 promoted the expression of miR-223-3p and that miR-223-3p bound to lncRNA AC040162.3 and the NLRP3 binding site. In addition, the protective effects of LncRNA AC040162.3 silencing in HCV-infected MIN6 cells were reversed by overexpression of NLRP3 or silencing of miR-223-3p.

**Conclusion:**

Silencing of lncRNA AC040162.3 alleviates the process of HCV-induced T2DM by governing the miR-223-3p/NLRP3 axis.

## 1. Introduction

Diabetes mellitus (DM), distinguished by high blood sugar, is among the most common endocrine diseases [1, 2]. It is estimated that over 4.51 million people have diabetes worldwide [3]. More than 90% of people with diabetes have type 2 diabetes mellitus (T2DM) [4]. The increased incidence of T2DM is associated with several risk factors, including obesity, population aging, and viral infection [5–7]. Prior studies found that an increased prevalence of T2DM is associated with chronic hepatitis C virus (HCV) infection [8]. Furthermore, insulin resistance (IR) and T2DM are the most common endocrine disorders in HCV-infected individuals. HCV accelerates the progression of T2DM compared with non-HCV-infected T2DM patients [9]. IR is the leading cause of overall mortality in HCV cirrhosis with IR or diabetes [10].

HCV has an intimate association with T2DM [11]. Patients with T2DM–HCV had remarkably higher values for lower diastolic and systolic blood pressure and Low Density Lipoprotein (LDL)-cholesterol than patients with T2DM [12–14]. Long noncoding RNAs (lncRNAs) are a class of functional RNA molecules that do not have protein coding ability but have functions such as transcriptional regulation, chromosome modification, and posttranscriptional processing [15]. Some lncRNAs are involved in the incidence of T2DM and HCV, and increasing evidence suggests this [16–18]. LncRNA NONRATT021972 siRNA normalized abnormal hepatic glucokinase function in T2DM rats through the Serine/threonine protein kinase AKT signaling pathway [19]. LncRNA MALAT1 regulates Reactive Oxygen Species (ROS) and insulin production in male mice [20]. LncRNA UCA1 regulates antiviral response and HCV replication via the miR-145-5p/SOCS7/IFN axis [21]. HCV induces the downregulation of LncRNA Linc-Pint expression to evade the innate immune system [22]. LncRNA KCNQ1OT1 promotes HCV-induced apoptosis by mediating the miR-223-3p/NLRP3 axis [23].

Our previous study found that the expression of a new lncRNA, AC040162.3, in HCV–T2DM increased significantly. This study mainly explored the mechanism by which lncRNA AC040162.3 affects HCV–T2DM.

## 2. Materials and Methods

### 2.1. Clinical Samples

Serum samples were collected from 20 HCV–T2DM patients and 20 T2DM patients. These patients were from the First People's Hospital of Yunnan Province between January 2020 and November 2021. Patients were selected for the present study according to the following inclusion criteria: no family history of diabetes and body mass index <40. Patients meeting the following exclusion criteria were excluded: (i) other types of diabetes, (ii) acute cerebrovascular disease, (iii) severe infections, and (iv) recent surgery. All uses of human samples were approved by the ethics committee of the First People's Hospital of Yunnan Province (KHLL2020-KY059), and all participants provided written informed consent forms.

### 2.2. Cell Culture

The mouse insulinoma cell line (MIN) and human hepatoma cell line Huh7.5.1 were purchased from the American Type Culture Collection. Cell culture was performed according to a previous method [23]. HCV (J6/JFH-1 strain) expansion cultures have been described previously [23]. The MIN6 cells were infected with HCV at a multiplicity of infection (MOI) of 1 and then cultured for 72 hours to obtain HCV-infected MIN6 cells.

### 2.3. Cell Transfection

The pcDNA3.1-lnc-AC040162.3 and pcDNA3.1-NLRP3 plasmids were constructed by subcloning the lnc-AC040162.3 and NLRP3 sequences into the pcDNA3.1 vector (Invitrogen, USA). Moreover, small interfering RNA targeting AC040162.3 (si-AC040162.3) was purchased from GenePharma. Cells were transfected with Lipofectamine 3000 (Invitrogen, USA) according to the manufacturer's protocol. In addition, Z-DEVD-FMK (caspase-3 inhibitor) and MCC950 (NLRP3 inhibitor) were used to culture with MIN6 cells according to the manufacturer's instructions.

### 2.4. Flow Cytometry

After transfection, cells (1 × 10^6^ cells/mL) were collected and digested with 0.25% trypsin. Based on the manufacturer's instructions, apoptosis of MIN6 cells was determined with an Annexin V-FITC/PI kit (Multi Sciences, China). Finally, apoptosis was measured by flow cytometry (Beckman, USA).

### 2.5. TUNEL

According to the TdT-mediated dUTP Nick End Labeling (TUNEL) kit instructions, the cells were incubated with TUNEL detection solution for 1 hour, followed by washing three times with phosphate buffer saline (PBS). Subsequently, the cells were counterstained with 4′,6-diamidino-2-phenylindole (DAPI) (1 mg/mL) for 10 minutes. Finally, the plate was mounted with anti-fluorescence quenching liquid and observed using an inverted microscope (Olympus, Tokyo, Japan).

### 2.6. RT-qPCR

According to the kit instructions, total RNA was extracted using TRIzol reagent. Total RNA was then reverse-transcribed to cDNA. Subsequently, quantitative PCR was performed on a 7500 real-time PCR system. The fluorescence quantitative primer sequences are listed in Table 1.

### 2.7. Western Blot

The protein concentration was determined after lysing MIN6 cells to extract total protein. After isolation, transfer blockade and incubation with the corresponding primary antibodies against Cleaved caspase-3, Caspase-1, Bcl-2, Bax, caspase-1, IL-18, IL-1*β*, and GAPDH were performed. Subsequently, the membrane was incubated with the secondary antibody for 1 hour. Detection was performed after adding the ECL Chemiluminescence Kit (Thermo Fisher Scientific, USA) and quantified by ImageJ.

### 2.8. Luciferase Reporter Assay

The pmirGLO dual-luciferase vector (AC040162.3-WT, AC040162.3-MUT, NLRP3-WT, NLRP3-MUT) and miR-223-3p mimic were cotransfected into HEK293T cells. After 24 hours of cocultivation, luciferase activities were investigated with a dual-luciferase assay system (Promega, USA).

### 2.9. ELISA

The cells were collected, and the supernatants were obtained by centrifugation at 1000 rpm for 5 minutes. Subsequently, insulin concentrations were measured using an insulin Enzyme-Linked Immunosorbent Assay (ELISA) kit.

### 2.10. Methyl Thiazolyl Tetrazolium Assay

Methyl thiazolyl tetrazolium (MTT) experiments were performed according to the kit instructions (Beyotime, China), and an automated enzyme labeling instrument was used to detect the optical density (OD) of each well at a wavelength of 490 nm.

### 2.11. Data Analysis

GraphPad Prism 8.0 was used to analyze the experimental data and plot the graphs. The analysis results are expressed as the mean ± SD. One-way analysis of variance and *t* tests were used, and *P* < 0.05 was considered statistically significant.

## 3. Results

### 3.1. Differential Expression of LncRNA AC040162.3, miRNA-223-3p and NLRP3

**Figure 1:**
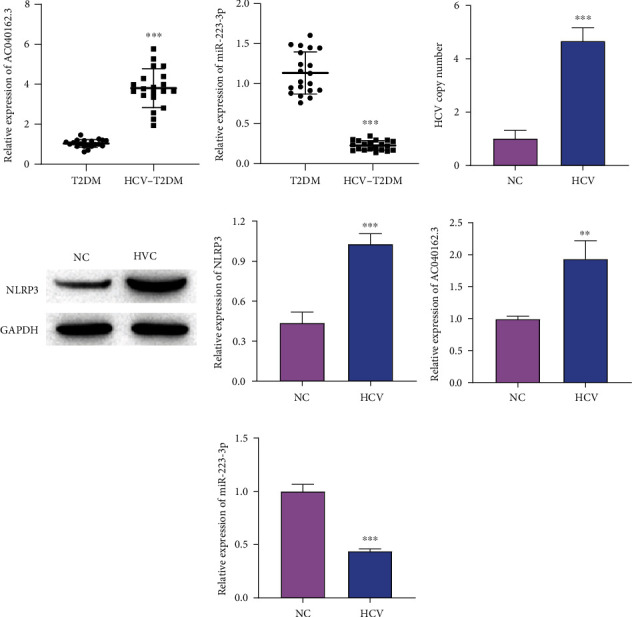
The expression of LncRNA AC040162.3, miRNA-223-3p, and NLRP3 in clinical samples and MIN6. (a and b) Levels of LncRNA AC040162.3 and miRNA-223-3p were evaluated by RT-qPCR. (c) The HCV copy number was detected by RT-qPCR. (d) Levels of NLRP3 were detected by Western blotting. (e and f) Levels of LncRNA AC040162.3 and miRNA-223-3p were quantified by RT-qPCR. ∗∗*P* < 0.01, ∗∗∗*P* < 0.001, vs. T2DM or NC.

To investigate the roles of LncRNA AC040162.3 and miRNA-223-3p in HCV–T2DM, we investigated the expression of LncRNA AC040162.3 and miRNA-223-3p in HCV–T2DM and non-HCV–T2DM patients (*n* = 20). LncRNA AC040162.3 expression levels were higher in HCV–T2DM patients than in non-HCV–T2DM patients (Figure 1(a)). In contrast, miRNA-223-3p expression was markedly reduced in HCV–T2DM patients (Figure 1(b)). To characterize the role of LncRNA AC040162.3, miRNA-223-3p, and NLRP3 in HCV–MIN6 cells, we performed Real Time Quantitative PCR (RT-qPCR) to quantify the copy number of HCV. The copy number of MIN6 cells was markedly increased after HCV infection, indicating the successful establishment of the HCV cell model (Figure 1(c)). We then determined the expression levels of lncRNA AC040162.3 and miRNA-223-3p by RT-qPCR. Compared with the NC group, the lncRNA AC040162.3 had a higher expression level, and the miRNA-223-3p expression level was lower in the HCV group (Figures 1(e) and 1(f)). Moreover, the expression of NLRP3 was markedly increased in the HCV group (Figure 1(d)). Therefore, we speculated that lncRNA AC040162.3, miRNA-223-3p, and NLRP3 might be involved in HCV–T2DM.

### 3.2. LncRNA AC040162.3 Affects Cell Proliferation, Apoptosis and Pyrolysis in HCV–MIN6

**Figure 2:**
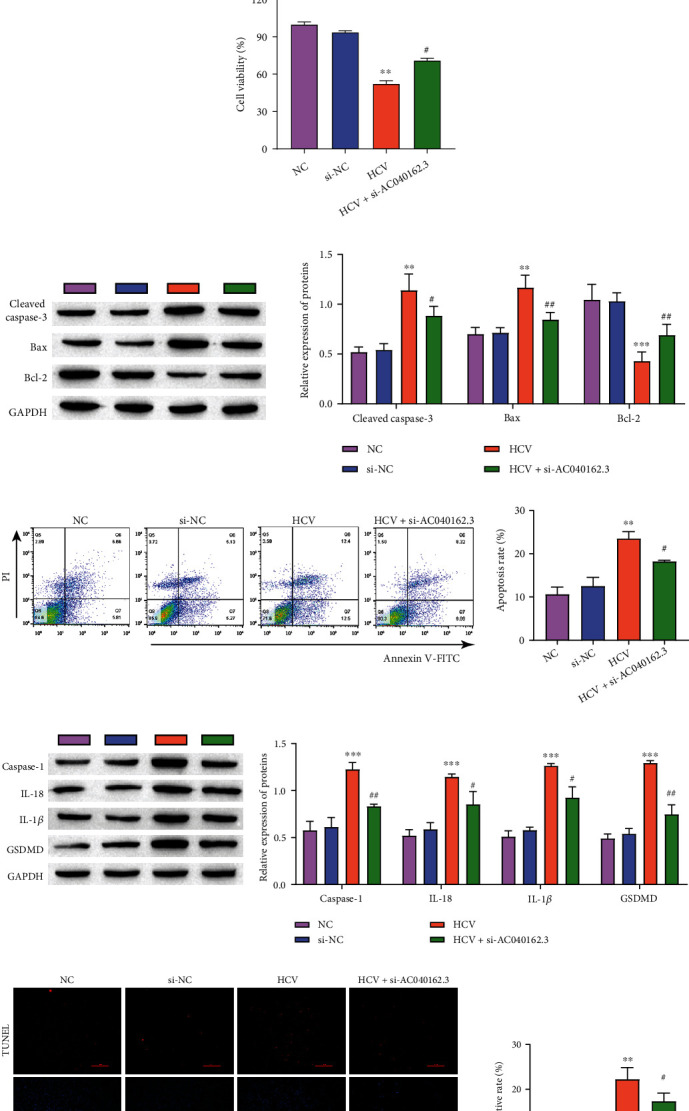
LncRNA AC040162.3 affects cell proliferation, apoptosis, and pyrolysis in HCV–MIN6. (a) The relative expression of LncRNA AC040162.3 in MIN6 cells transfected with si-AC040162.3-1, si-AC040162.3-2, and si-AC040162.3-3. (b) Insulin concentrations were measured by ELISA. (c) MTT assay was used to determine the cell proliferation rate. (d) The levels of Cleaved caspase-3, Bax, and Bcl_2_ were detected by Western blotting. (e) Flow cytometry analysis of cell death. (f) The levels of Caspase-1, IL-18, and IL-1*β* were detected by Western blotting. (g) TUNEL-determined cell pyrolysis (scale bar = 100 *μ*m). ∗∗*P* < 0.01, ∗∗∗*P* < 0.001, vs. si-NC. #*P* < 0.05, ##*P* < 0.01, ###*P* < 0.001, vs. HCV.

To characterize the role of LncRNA AC040162.3 in HCV infection, we first studied the influence of silencing LncRNA AC040162.3 on the proliferation, apoptosis, and pyrolysis of MIN6 cells. Transfection efficiency analysis showed that si-AC040162.3-1 had better interference efficiency than si-AC040162.3-2 and si-AC040162.3-3 (Figure 2(a)), so si-AC040162.3 1 was used for the following experiments. ELISA analysis showed that the insulin secretion of the si-NC group was similar to that of the NC group, HCV inhibited insulin secretion, while the silencing of LncRNA AC040162.3 significantly increased insulin secretion (Figure 2(b)). HCV treatment inhibited cell viability, and knocking down lncRNA AC040162.3 improved cell viability (Figure 2(c)). The protein expression of the apoptosis-related proteins Cleaved caspase-3 and Bax was considerably upregulated in the HCV group, while the expression of Cleaved caspase-3 and Bax was inhibited by knocking down the lncRNA AC040162.3 (Figure 2(d)). Similar results were obtained by flow cytometry to detect apoptosis (Figure 2(e)). In addition, we used TUNEL and Western blotting to examine the effect of LncRNA AC040162.3 on cell pyrolysis. Pyroptosis-related molecules, Caspase-1, IL-18, IL-1*β* and GSDMD, were highly expressed in the HCV group, and silencing of LncRNA AC040162.3 significantly reduced the expression of these proteins (Figure 2(f)). TUNEL staining showed similar results (Figure 2(g)). These results showed that LncRNA AC040162.3 regulated HCV-infected proliferation, apoptosis, and pyrolysis of MIN6 cells. Silencing of LncRNA AC040162.3 inhibited proliferation, apoptosis, and pyrolysis of MIN6 cells induced by HCV infection to increase insulin secretion.

### 3.3. Caspase-3 Influences Cell Proliferation, Apoptosis and Pyrolysis in HCV–MIN6

**Figure 3:**
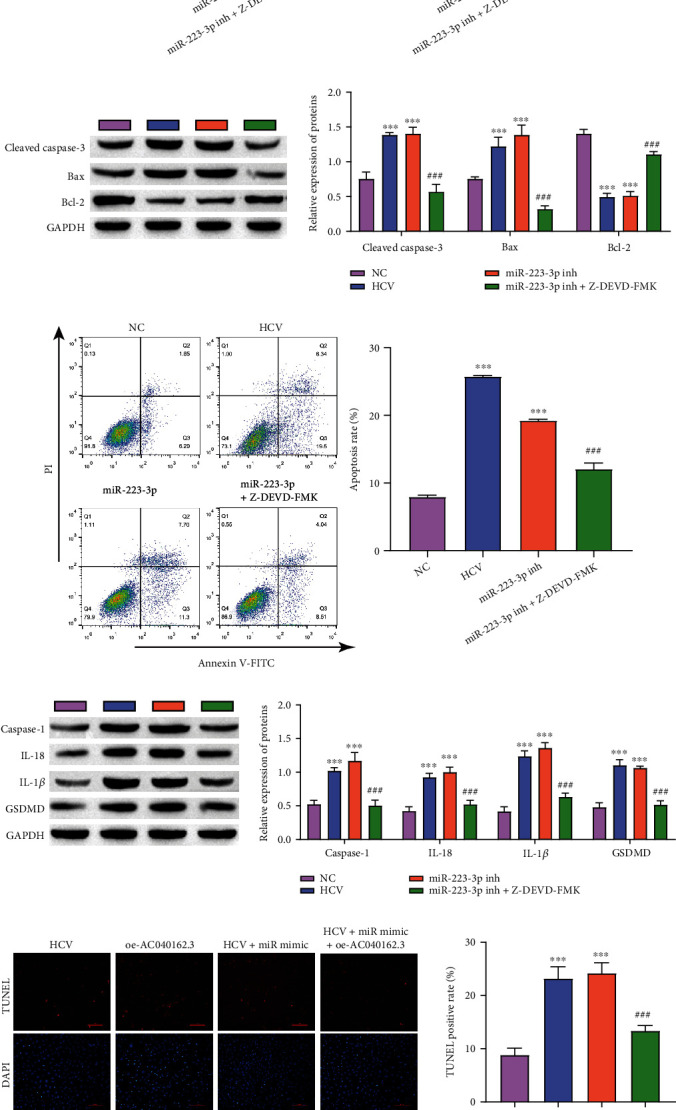
Caspase-3 influences cell proliferation, apoptosis, and pyrolysis in HCV–MIN6. (a) Insulin concentrations were detected by ELISA. (b) MTT assay was used to determine the cell proliferation rate. (c) The levels of Cleaved caspase-3, Bax, and Bcl_2_ were detected by Western blotting. (d) Flow cytometry analysis of cell death. (e) The levels of Caspase-1, IL-18, and IL-1*β* were detected by Western blotting. (f) TUNEL-determined cell pyrolysis (scale bar = 100 *μ*m). ∗∗∗*P* < 0.001, vs. NC. #*P* < 0.05, ###*P* < 0.001, vs. miR-223-3p inh.

In this study, we checked whether the caspase-3 inhibitor influenced the effect of miR-223-3p in the cell model and whether pyroptosis still functioned under caspase-3 inhibitor treatment. Z-DEVD-FMK (a caspase-3 inhibitor) was used to investigate the role of miR-223-3p. ELISA analysis showed that the insulin secretion of the HCV group was similar to that of the miR-223-3p inhibitor group, while Z-DEVD-FMK significantly increased insulin secretion (Figure 3(a)). HCV and miR-223-3p inhibitor treatment inhibited cell viability, and Z-DEVD-FMK improved cell viability (Figure 3(b)). In addition, the protein expression of the apoptosis-related proteins, Cleaved caspase-3 and Bax, was considerably upregulated in the HCV and miR-223-3p inhibitor groups, while the expression of Cleaved caspase-3 and Bax was inhibited by Z-DEVD-FMK (Figure 3(c)). Similar results were obtained by flow cytometry (Figure 3(d)). In addition, we used TUNEL and Western blotting to detect the effect of Z-DEVD-FMK on cell pyroptosis. The pyroptosis-related molecules, Caspase-1, IL-18, IL-1*β*, and GSDMD, were highly expressed in the HCV and miR-223-3p inhibitor groups, and Z-DEVD-FMK significantly reduced the expression of these proteins (Figure 3(e)). TUNEL staining showed similar results (Figure 3(f)). These results showed that Caspase-3 regulated HCV-infected proliferation, apoptosis, and pyrolysis of MIN6 cells and was related to miR-223-3p.

### 3.4. The Targeting Relationship between miR-223-3p and LncRNA AC040162.3 or LncRNA AC040162.3 and NLRP3

**Figure 4:**
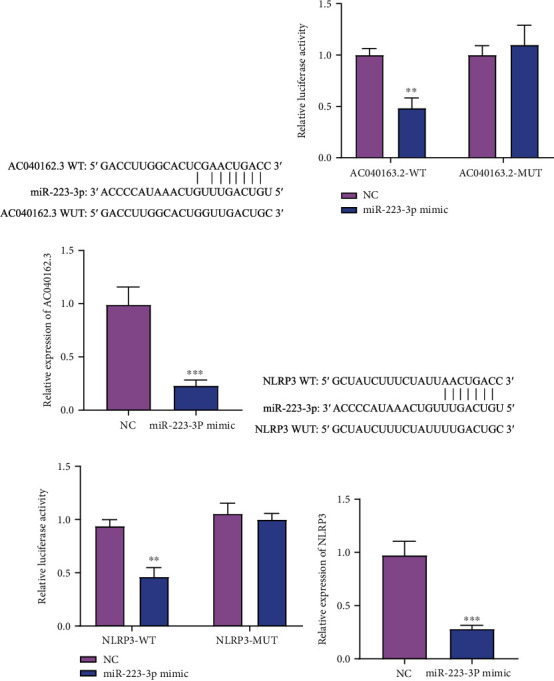
Identification of targeting relationships. (a) Schematic diagram of the putative binding sites of LncRNA AC040162.3 and miR-223-3p. (b) Validation of the targeting relationship between lncRNA AC040162.3 and miR-223-3p. (c) RT-qPCR analysis of LncRNA AC040162.3. (d) Schematic diagram of the miR-223-3p putative binding sites and mutant site in the 3′-UTR of NLRP3 predicted by bioinformatics. (e) Validation of the targeting relationship between miR-223-3p and NLRP3. (f) RT-qPCR analysis of NLRP3. WT: wild-type; MUT: mutant-type. ∗∗*P* < 0.01, ∗∗∗*P* < 0.001, vs. NC.

To confirm whether LncRNA AC040162.3 and NLRP3 can bind to miR-223-3p, we cotransfected miR-223-3p with LncRNA AC040162.3 or NLRP3 (WT or Mut) into HEK293T cells. The binding and mutation sites in the LncRNA AC040162.3 3′ UTR and NLRP 3′ UTR are shown in Figures 4(a) and 4(d). Further verification by dual-luciferase experiments showed that LncRNA AC040162.3 and NLRP3 could bind to miR-223-3p (Figures 4(b) and 4(e)). Furthermore, the expression of lncRNA AC040162.3 and NLRP3 was significantly reduced after transfection with miR-223-3p mimics (Figures 4(c) and 4(f)). Therefore, we speculate that lncRNA AC040162.3 participates in the molecular mechanism of HCV-induced deterioration of T2DM through the miR-223-3p/NLRP3 axis.

### 3.5. LncRNA AC040162.3 Promotes HCV-Induced T2DM Deterioration through miR-223-3p

**Figure 5:**
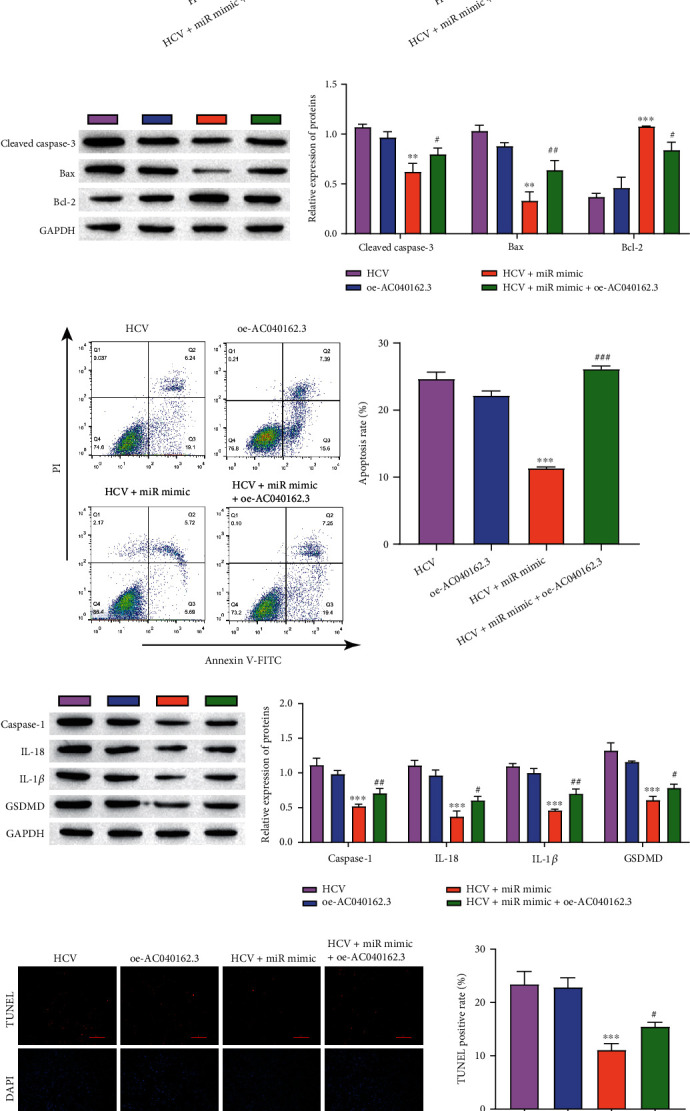
LncRNA AC040162.3 promotes HCV-induced T2DM deterioration through miR-223-3p. (a) Insulin concentrations were detected by ELISA. (b) MTT assay was used to determine the cell proliferation rate. (c) The levels of Cleaved caspase-3, Bax, and Bcl_2_ were detected by Western blotting. (d) Flow cytometry analysis of cell death. (e) The levels of Caspase-1, IL-18, and IL-1*β* were detected by Western blotting. (f) TUNEL was used to determine cell death (scale bar = 100 *μ*m). ∗∗*P* < 0.01, ∗∗∗*P* < 0.001, vs. HCV. #*P* < 0.05, ##*P* < 0.01, ###*P* < 0.001, vs. HCV + miR mimic.

In this study, we explored the molecular mechanism by which lncRNA AC040162.3 regulates HCV-induced T2DM deterioration through miR-223-3p. Insulin secretion in the miR-223-3p mimic group was markedly increased, while insulin secretion was significantly decreased after overexpression of lncRNA AC040162.3 (Figure 5(a)). The MTT assay results proved that the miR-223-3p mimic increased cell viability, while lncRNA AC040162.3 was able to inhibit this effect (Figure 5(b)). Overexpression of miR-223-3p downregulated the expression of Cleaved caspase-3 and Bax, and upregulated the expression of Bcl_2_ in MIN6 cells, whereas overexpression of lncRNA AC040162.3 reversed this effect (Figure 5(c)). Similar results were obtained in the detection of apoptosis by flow cytometry (Figure 5(d)). Furthermore, the effect of the lncRNA AC040162.3/miR-223-3p axis on the pyrolysis of MIN6 cells was examined by Western blot and TUNEL assays. The expression of Caspase-1, IL-18, IL-1*β* and GSDMD was significantly inhibited by the miR-223-3p mimic, and overexpression of lncRNA AC040162.3 reversed this inhibition (Figure 5(e)). TUNEL results showed that the pyrolysis rate of cells was markedly reduced in the miR-223-3p mimic group, while cotransfection of LncRNA AC040162.3 and miR-223-3p significantly reversed this inhibition (Figure 5(f)). In conclusion, our study indicates that lncRNA AC040162.3 promotes HCV-induced T2DM deterioration through miR-223-3p.

### 3.6. LncRNA AC040162.3 Promotes HCV-Induced T2DM Deterioration through the miR-223-3p/NLRP3 Axis

**Figure 6:**
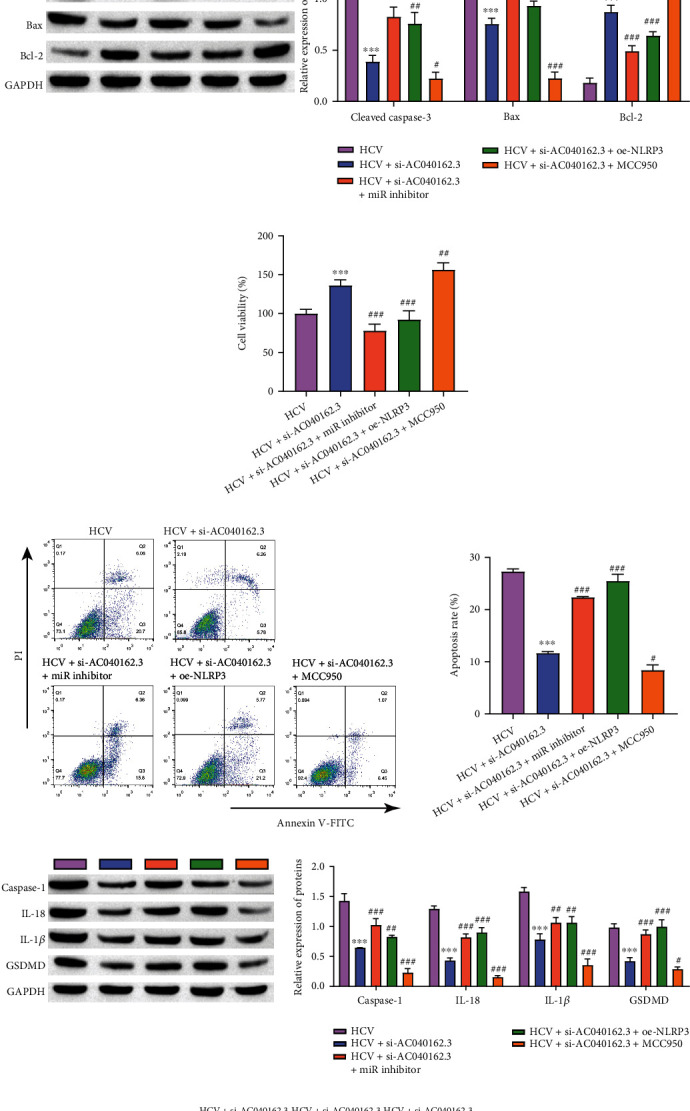
LncRNA AC040162.3 promotes the molecular mechanism of HCV-induced T2DM deterioration through miR-223-3p. (a) Insulin concentrations were detected by ELISA. (b) Protein levels of Cleaved caspase-3, Bax and Bcl2 were detected by Western blot. (c) MTT assay was used to determine the cell proliferation rate. (d) Flow cytometry analysis of cell apoptosis. (e) Protein levels of Caspase-1, IL-18, and IL-1*β* were detected by Western blot. (f) TUNEL was used to determine cell death (scale bar = 100 *μ*m). ∗∗∗*P* < 0.001, vs. HCV. #*P* < 0.05, ##*P* < 0.01, ###*P* < 0.001, vs. HCV + si-AC040162.3.

Finally, we assessed the effects of LncRNA AC040162.3, miR-223-3p, and NLRP3 on the progression of HCV-induced diabetes. ELISAs showed that silencing of LncRNA AC040162.3 promoted insulin secretion, while simultaneous transfection of si-LncRNA AC040162.3 and miR-223-3p inhibitor or si-LncRNA AC040162.3 and oe-NLRP3 inhibited this promotion. MCC950 (NLRP3 inhibitor) further promoted insulin secretion (Figure 6(a)). Western blot analysis further revealed that lncRNA AC040162.3 downregulated the expression of the apoptosis markers Cleaved caspase-3 and Bax, and upregulated Bcl_2_ expression. However, cotransfection with si-LncRNA AC040162.3 and miR-223-3p inhibitor or si-LncRNA AC040162.3 and oe-NLRP3 inhibited this change, and MCC950 further inhibited cell apoptosis (Figure 6(b)). MTT results proved that silencing of LncRNA AC040162.3 increased cell viability, while cells transfected with si-LncRNA AC040162.3 and miR-223-3p inhibitor or si-LncRNA AC040162.3 and oe-NLRP3 showed significantly reduced cell proliferation, MCC950 further promoted cell viability (Figure 6(c)). Further tests by flow cytometry showed similar results (Figure 6(d)). Western blot analysis further showed that silencing lncRNA AC040162.3 downregulated the expression of the pyroptosis markers Caspase-1, IL-18, IL-1*β* and GSDMD. However, cotransfection with si-LncRNA AC040162.3 and miR-223-3p inhibitor or si-LncRNA AC040162.3 and oe-NLRP3 inhibited this change, and MCC950 further inhibited cell pyrolysis (Figure 6(e)). TUNEL results demonstrated similar results (Figure 6(f)). Thus, these results confirmed that lncRNA AC040162.3 promotes HCV-induced T2DM deterioration through the miR-223-3p/NLRP3 axis.

## 4. Discussion

T2DM and HCV infection are two major public health problems that cause financial burdens worldwide [24, 25]. Many clinical epidemiological studies have reported that HCV infection has been associated with diabetes since 1994 [26]. Furthermore, epidemiological studies have demonstrated that HCV infection is related to T2DM and IR [27]. Despite extensive research on them, the underlying mechanism behind T2DM in HCV patients is still lacking. Recently, the cytopathic effect of HCV by affecting the level of pancreatic islet cells has been demonstrated [28]. Related studies have shown that lncRNAs have a great influence on many diseases, such as T2DM and HCV infection [29, 30]. LncRNAs affect many biological processes, including cell migration, proliferation, and apoptosis [31]. This study found that lncRNA AC040162.3 expression was higher in HCV–T2DM patients than in non-HCV–T2DM patients. In contrast, miRNA-223-3p expression was markedly reduced in HCV–T2DM patients, which indicates that LncRNA AC040162.3 and miRNA-223-3p might be involved in HCV–T2DM.

MicroRNAs (miRNAs), approximately 20 nt in length, are small noncoding single-stranded RNA molecules that regulate target genes by binding to the 3′ untranslated region (3′ UTR) [32]. MiRNAs and lncRNAs are essential participants in the biology of many diseases. Wang found that natural killer cell-derived exosomal miR-1249-3p attenuates IR and inflammation in mouse models of T2DM [33]. Expression of miRNA-29 in pancreatic *β* cells promotes inflammation and diabetes via TRAF3 [34]. Our study found that the silencing of LncRNA AC040162.3 after HCV infection can reduce MIN6 cell apoptosis and pyrolysis and promote proliferation. Notably, this inhibition was reversed after inhibiting the expression of miR-223-3p. In addition, this experiment demonstrated a targeting relationship between lncRNA AC040162.3 and miR-223-3p. Therefore, this study shows that lncRNA AC040162.3 has an essential influence on T2DM provoked by HCV by targeting miR-223-3p.

NLRP3 has been widely studied in inflammation prognosis and progression of T2DM [35]. In recent years, multiple studies have shown that NLRP3 is activated in HCV-induced T2DM, triggering the proliferation of pancreatic MIN6 cells, apoptosis, and pyroptosis, leading to impaired pancreatic islet cell function and triggering T2DM [36, 37]. In addition, miRNA-223 has been shown to be a key regulator of NLRP3 inflammasome activity [23, 38]. Therefore, we studied the potential molecular mechanism of LncRNA AC040162.3 through the miR-223-3p/NLRP3 axis to promote HCV-induced T2DM deterioration. The results showed that the silencing of LncRNA AC040162.3 after HCV infection can reduce the apoptosis and pyrolysis of MIN6 cells and promote proliferation, while the simultaneous transfection of si-AC040162.3 and oe-NLRP3 can reverse this inhibitory effect. MCC950 further reduced the apoptosis and pyrolysis of MIN6 cells. In addition, this study demonstrated that miR-223-3p could target and regulate NLRP3. Further results confirmed that lncRNA AC040162.3 affected cell proliferation, pyroptosis, and apoptosis in HCV-infected MIN6 cells through the miR-223-3p/NLRP3 axis.

## 5. Conclusion

Collectively, our study reports that LncRNA AC040162.3 promotes apoptosis and pyrolysis of HCV-infected MIN6 cells through the miRNA-223-3p/NLRP3 axis, inhibits proliferation, reduces insulin production, and accelerates the onset and progression of T2DM. This indicates that lncRNA AC040162.3 may serve as a potential target of HCV-induced T2DM.

## Figures and Tables

**Table 1:** Primer sequences.

Gene	Forward 5′–3′	Reverse 5′–3′
*LncRNA AC040162.3*	*AAAACGAGTGCTTGGCGTTC*	*GCCCTCCAGATAGACGAAGC*
*miR-223-3p*	*CGCUAUCUUUCUAUUAUGACUCCAUAA*	*GTGGAGATCCTAGGTTTCTCTG*
*NLRP3*	*TTGGATCAGGGAGTTGGAAG*	*CAGGATCTCATTCTCTTGGATC*
*U6*	*GCTTCGGCAGCACATATACTAAAAT*	*CGCTTCACGAATTTGCGTGTCAT*
*GAPDH*	*CTTTGGTATCGTGGAAGGACTC*	*GTAGAGGCAGGGATGATGTTCT*

## Data Availability

The datasets used and/or analyzed during the current study are available from the corresponding author upon reasonable request.
